# Reduction of the *Fusarium* Mycotoxins: Deoxynivalenol, Nivalenol and Zearalenone by Selected Non-Conventional Yeast Strains in Wheat Grains and Bread

**DOI:** 10.3390/molecules27051578

**Published:** 2022-02-27

**Authors:** Izabela Podgórska-Kryszczuk, Ewa Solarska, Monika Kordowska-Wiater

**Affiliations:** 1 Department of Analysis and Food Quality Assessment, University of Life Sciences in Lublin, Skromna 8, 20-704 Lublin, Poland; izabela.podgorska-kryszczuk@up.lublin.pl; 2 Department of Biotechnology, Microbiology and Human Nutrition, University of Life Sciences in Lublin, Skromna 8, 20-704 Lublin, Poland; ewa.solarska@up.lublin.pl

**Keywords:** *Fusarium culmorum*, *Fusarium graminearum*, *Fusarium poae*, yeast, mycotoxin, deoxynivalenol, nivalenol, zearalenone

## Abstract

Mycotoxins, toxic secondary metabolites produced by fungi, are important contaminants in food and agricultural industries around the world. These toxins have a multidirectional toxic effect on living organisms, causing damage to the kidneys and liver, and disrupting the functions of the digestive tract and the immune system. In recent years, much attention has been paid to the biological control of pathogens and the mycotoxins they produce. In this study, selected yeasts were used to reduce the occurrence of deoxynivalenol (DON), nivalenol (NIV), and zearalenone (ZEA) produced by *Fusarium culmorum*, *F. graminearum*, and *F. poae* on wheat grain and bread. In a laboratory experiment, an effective reduction in the content of DON, NIV, and ZEA was observed in bread prepared by baking with the addition of an inoculum of the test yeast, ranging from 16.4% to 33.4%, 18.5% to 36.2% and 14.3% to 35.4%, respectively. These results indicate that the selected yeast isolates can be used in practice as efficient mycotoxin decontamination agents in the food industry.

## 1. Introduction

Fungi of the genus *Fusarium*, due to their pathogenicity and high toxigenicity, generate huge problems in agriculture [1]. The most mycotoxigenic species are *F. culmorum* and *F. graminearum* [2], but other species, such as *F. poae, F. sporotrichioides, F. oxysporum,* and *F. verticillioides*, also pose a serious threat [3]. These fungi produce several mycotoxins, including moniliformin (MON), fumonisins (FUM), beauvericin (BEA), ZEA, and trichothecenes, which show strong and varied toxic effects [4]. One species of the pathogen can produce more than one toxin, and one toxin can be synthesized by several species of fungi, therefore, the presence of various mycotoxins in a given raw material should be expected, and the possibility of interactions between them, including both synergism and antagonism, should be taken into account [5]. From the point of view of the prevalence of occurrence and harmfulness to living organisms and to economy, the most important of the *Fusarium* mycotoxins are trichothecenes (DON, NIV, diacetoxyscirpenol (DAS), T-2 and HT-2 toxins), ZEA, and FUM [6]. Trichothecenes are the most numerous groups of *Fusarium* mycotoxins. They include over 180 compounds found in nature [7]. At the cellular level, trichothecenes inhibit protein biosynthesis, reduce enzyme activity, disturb cell division, affect the permeability of cytoplasmic membranes, induce chromosomal aberrations, and disrupt the course of the cell cycle [8,9,10]. DON, due to its toxicity and widespread occurrence, is considered to be the most significant mycotoxin contaminating cereal grains intended for food purposes [11,12]. ZEA is characterized by relatively low acute toxicity, but long-term exposure to this compound causes fertility disorders, leading to hyperestrogenism [13].

Wheat grain is a basic raw material used in human nutrition and, at the same time, one that is the most exposed to contamination by toxicogenic fungi. The presence and concentration of *Fusarium* mycotoxins in cereals depend on many factors, including the toxicogenic abilities of a specific fungal species, the species of the infected plant, climatic conditions, and the presence of competitive microflora [14,15,16]. Fungi may also produce higher levels of mycotoxins when growing under stressful conditions and in the presence of chemical plant protection products [17,18]. The presence of pathogenic fungi in crops and their contamination with mycotoxins is a key threat and has a significant impact on food and feed safety, but also has serious economic effects and affects international trade [19]. For this reason, minimizing mycotoxin contamination has become a priority for scientists and organizations, including the World Health Organization (WHO), Food and Agriculture Organization of the United Nations (FAO), and European Food Safety Authority (EFSA). Reduction of the presence of mycotoxins in grain is extremely important because, if contaminated flour is used for baking, the content of these toxins in the bread will be similar to the amount in the flour [20,21]. Moreover, in extruded wheat products, the content of DON and NIV may increase after processing [22].

Unfortunately, mycotoxins are resistant to sterilization and pasteurization and most physicochemical factors, so their elimination from the product is very difficult [23]. Given the limitations of this method of decontamination, biological methods using microorganisms or their enzymes are becoming the focus of more research [16]. Nowadays, high hopes are pinned on the formulation of new antimicrobial starter cultures, which would significantly improve food quality and safety. Yeast cells and their metabolites also hold great potential for minimizing the economic losses caused by pathogenic fungi. Selected strains with appropriate technological features could reduce the content of toxins, increasing the safety of the final product [24,25]. Identification of yeast strains that synthesize antifungal compounds and have the ability to lower the content of toxins would make it possible to extend the shelf life of food products and improve their quality.

In this study, seven yeast strains were used to reduce the production of mycotoxins by *F. culmorum, F. graminearum* and *F. poae* on wheat grain. The yeasts were selected on the basis of their ability to limit the mycelial growth of pathogens, and the results are presented in a previous work [26]. The aim of this study was to select yeasts which, when added to dough, have the potential to reduce *Fusarium* mycotoxins in grain products.

## 2. Results

### 2.1. Determination of Mycotoxin Contents in Wheat Grain

*F. culmorum* produced the highest amount of DON, followed by NIV; the mean content of these toxins in the control sample was 1184.60 µg/kg and 917.4 µg/kg, respectively. The content of ZEA in the control was 241.8 µg/kg. In some variants of the experiment, after incubation of *F. culmorum* with yeasts, the DON and ZEA contents were greater than in the control sample, in which fungi alone were grown on the cereal. *C. saturnus* (K10) and *R. glutinis* (E20) increased the production of mycotoxins by *F. culmorum* in wheat grain but received results do not differ significantly (*p* < 0.05) compared to the control. The content of DON in the samples with these isolates was 1218.3 µg/kg and 1186.2 µg/kg, respectively, and the content of ZEA was 246 µg/kg and 247.8 µg/kg, respectively (Table 1).

**Figure 1:**
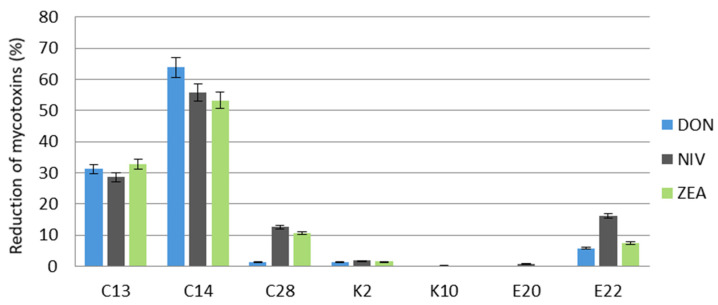
Reduction of deoxynivalenol, nivalenol, and zearalenone produced by *F. culmorum* in wheat grain after a 14-day incubation with the test yeasts. C13, C14, C28, K2, K10, E20, E22—code numbers of the yeast species acc. to Table 1. Error bar represents the standard error.

The highest percentage reduction in the mycotoxins produced by *F. culmorum* was recorded after incubation with the yeast *C. fluviatilis* (C14): 63.9% for DON, 55.9% for NIV, and 53.3% for ZEA. The second most potent inhibitor of mycotoxin production was *C. shehatae* (C13), which reduced the content of DON by 31.2%, NIV by 28.7%, and ZEA by 32.8% (Figure 1).

In the control trial, *F. graminearum* growing on wheat grain produced the highest amount of DON at 1382.3 µg/kg. Additionally, the content of NIV was high at 932.2 µg/kg, while the content of ZEA was 273.3 µg/kg (Table 2).

**Figure 2:**
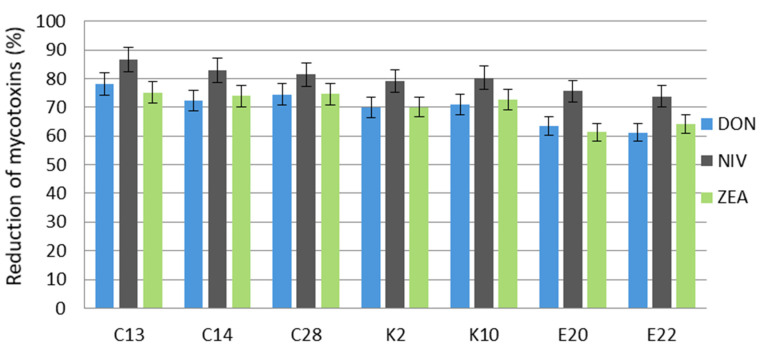
Reduction of deoxynivalenol, nivalenol, and zearalenone produced by *F. graminearum* in wheat grain after a 14-day incubation with the test yeasts. C13, C14, C28, K2, K10, E20, E22—code numbers of the yeast species acc. to Table 2. Error bar represents the standard error.

All the test yeasts reduced the occurrence of DON, NIV, and ZEA produced by *F. graminearum* to a significant extent, above 60%. In all trials, the test isolates most effectively reduced the NIV content, in the range from 73.8% to 86.6%. The amounts of DON and ZEA in wheat grain were also significantly lowered in all culture combinations, and the greatest reduction in the level of these toxins was caused by the yeast *C. shehatae* (C13) at 78.3% and 75.1%, respectively, followed by *C. tropicalis* (C28), which reduced the content of DON and ZEA by 74.5% and 74.6%, respectively (Figure 2).

*F. poae,* in the control sample, produced similar mean amounts of DON and NIV in wheat grain at 924.6 µg/kg and 971.3 µg/kg, respectively, and significantly lower amounts of ZEA at 106.0 µg/kg (Table 3). As in the case of *F. culmorum*, in some variants of the experiment, after incubation with yeasts, *F. poae* produced higher amounts of DON and ZEA than in the control sample but the received results do not differ significantly (*p* < 0.05) compared to the control. After 14 days of incubation of the yeast *C. carnescens* (E22) with *F. poae*, the DON content was 955.0 µg/kg. A higher content of ZEA in comparison to the control was found in the sample with the yeast *R. glutinis* (E20)—108.3 µg/kg.

**Figure 3:**
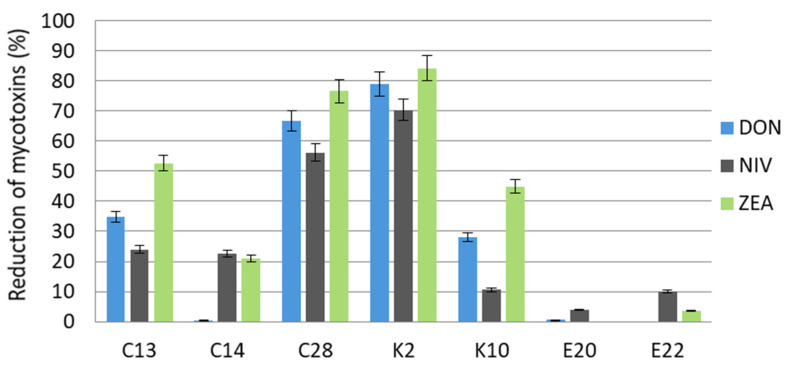
Reduction of deoxynivalenol, nivalenol, and zearalenone produced by *F. poae* in wheat grain after a 14-day incubation with the test yeasts. C13, C14, C28, K2, K10, E20, E22—code numbers of the yeast species acc. to Table 3. Error bar represents the standard error.

The highest reduction of the mycotoxins produced by *F. poae* was obtained with the use of the yeasts *M. guilliermondii* (K2) and *C. tropicalis* (C28). These yeasts, respectively, reduced the content of DON by 79.0% and 66.7%, NIV by 70.4% and 56.3%, and ZEA by 84.2% and 76.6%. On the other hand, *R. glutinis* (E20) and *C. carnescens* (E22) were the least efficient in reducing the contents of the three mycotoxins (Figure 3).

### 2.2. Determination of Biogenic Amines

The ability to produce biogenic amines was found in the yeasts *C. tropicalis* (C28) and *C. carnescens* (E22). These isolates were surrounded by a purple halo, which grew larger and darker over time (data not shown). The yeast species that did not produce biogenic amines discoloured the medium yellow due to glucose fermentation.

### 2.3. Determination of Mycotoxin Contents in Model Bread

**Figure 4:**
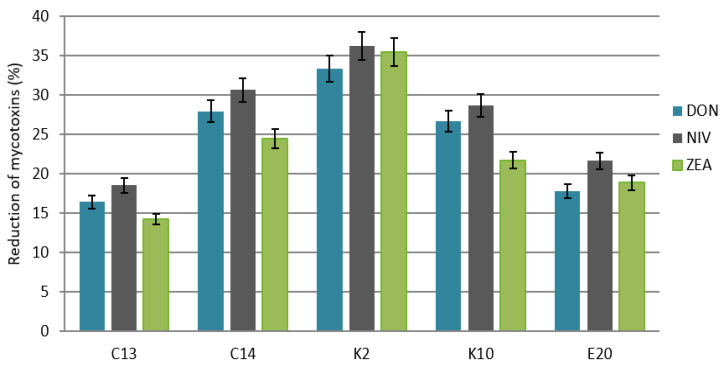
Reduction of deoxynivalenol, nivalenol, and zearalenone in wheat grain bread inoculated with *F. culmorum, F. graminearum, F. poae* and the test yeasts. C13, C14, K2, K10, E20—code numbers of the yeast species acc. to Table 4. Error bar represents the standard error.

The highest content of DON, at 1380.8 µg/kg, was found in the control bread. The concentrations of NIV and ZEA in the control sample were 933.5 µg/kg and 204.5 µg/kg, respectively (Table 4). It was observed that the addition of yeast inoculum to the dough reduced the contents of all the test mycotoxins in the model bread made from flour obtained from grain contaminated with fungi. The reductions in DON, NIV, and ZEA contents for each variant of the experiment are shown in Figure 4.

*M. guilliermondii* (K2) caused the greatest reduction in the content of the test mycotoxins in bread: 36.2% for NIV, 35.4% for ZEA, and 33.4% for DON, compared to the control. It was followed by *C. fluviatilis* (C14), which reduced the content of NIV by 30.6%, DON by 27.9%, and ZEA by 24.5%. The weakest reduction in the content of the mycotoxins in the examined bread was observed for *C. shehatae* (C13), which lowered the content of NIV by 18.5%, DON by 16.4%, and ZEA by 14.3%.

## 3. Discussion

In most cases of this study, the yeast effectively reduced the concentration of the mycotoxins produced by *F. culmorum*, *F. graminearum*, and *F. poae*. The level of reduction depended on the degree of inhibition of the pathogen’s mycelial growth. Limiting the growth of mycelium is important, not only for the proper development of the plant (including cereals) but for the improvement of the health and safety of the obtained grain and, consequently, the products made of it. In the previous work [26], we showed that the tested yeasts reduce spore germination and mycelium growth of *F. culmorum*, *F. graminearum*, and *F. poae*. The biocontrol yeasts strains may arrest fungal growth, reduce mycotoxin production or both. Undoubtedly, yeasts metabolites play a role in their mechanism of action although with a diversity of modes. The antagonistic mechanisms are various, including competition for nutrients and space, the production of antifungal volatile metabolites, and the production of lytic enzymes that degrade the cell wall of pathogens. Effective biocontrol strategy may be based on the combined use of multiple isolates with different mechanisms of action. Reported results has shown the potential of nonconventional yeast isolates to be biocontrol agents for selected fungi of the genus *Fusarium* and prevent deoxynivalenol, nivalenol and zearalenone accumulation.

The yeasts *C. shehatae* (C13), *C. fluviatilis* (C14), *M. guilliermondii* (K2), *C. saturnus* (K10), and *R. glutinis* (E20), which displayed the highest efficiency in reducing mycelial growth of tested pathogens, were also used in the model bread baking experiment. Unfortunately, some non-conventional strains can produce toxic metabolites such as biogenic amines (products of amino acid decarboxylation), which, if absorbed in high concentrations by the human body, act as neurotoxins [27]. They can cause adverse health symptoms such as blood pressure crises, headaches, stomach cramps, and diarrhoea [28]. Therefore, before new isolates are used in the food production process, they must be tested for safety. In this study the ability to produce biogenic amines was found in the yeasts *C. tropicalis* (C28) and *C. carnescens* (E22), and for that reason they were not used in the baking process. The addition of an inoculum of the test yeast to bread was found to effectively reduce the number of mycotoxins produced by the fungi. The yeast species that most efficiently reduced the production of NIV, ZEA (above 35%), and DON (above 33%) was *M. guilliermondii* (K2) isolated from the roots of conventionally-grown oat. Data from the literature reported the ability of these yeasts to reduce the number of other mycotoxins as well. Fu et al. [29] confirmed that *M. guilliermondii* could degrade patulin (PAT). Similar results were obtained by Chen et al. [30], who stated that *Candida guilliermondii* (teleomorph *M. guilliermondii*) could effectively reduce PAT content in culture medium. These yeasts are widely studied in various aspects due to their clinical importance, biotechnological applications and biological control potential [31].

In practice, relatively little attention is paid to the use of non-conventional yeast strains to ferment bread dough. The food industry mainly makes use of *Saccharomyces cerevisiae*, which has a high fermentation efficiency and affects the sensory properties of the product by producing the desired flavors [32,33]. In addition to the appropriate technological features, *S. cerevisiae* yeast has antagonistic properties against pathogenic bacteria and fungi [34] and the ability to reduce the content of toxins [35,36,37]. Soboleva et al. [38] found that *S. cerevisiae* RCAM 01730, when used in baking bread, inhibited the growth of undesirable bacteria. Armando et al. [39] demonstrated the ability of two yeast strains, *S. cerevisiae* RC008 and RC016, to inhibit the growth of *Aspergillus carbonarius* and *F. graminearum*, and reduce their production of the mycotoxins ochratoxin A (OTA), ZEA, and DON. Mozaffary et al. [40] confirmed that during dough fermentation baker’s yeast *S. cerevisiae* was able to reduce the amount of OTA in wheat flour by about 60%. Piotrowska and Żakowska [41] showed that baker’s yeast and lactic acid bacteria mixed culture were found to be efficient in toxin degradation, which could be explained by the synergistic relationship between these microorganisms in the baker’s sourdough. However, recent research suggests that there are also many other yeasts with the same properties as *S. cerevisiae* that qualify them as alternative baker’s yeast [42].

Literature reports demonstrate that there is a great potential for non-conventional yeast strains to be used in the modern baking industry in order to increase the aroma complexity of bread [43] and as potential leavening agents [44]. Non-conventional yeast show many other advantages such as freezing tolerance, amylase activity, and the ability to ferment complex sugars [43,45]. Specially selected yeast can also produce antifungal compounds [46,47] and have the ability to reduce the mycotoxin content [29,48], as demonstrated in this study. There are many examples in the literature that non-conventional yeast can effectively reduce the occurrence of mycotoxins. Medina-Córdova et al. [49] reported that the yeast *Debaryomyces hansenii*, isolated from the marine environment, showed the ability to inhibit the growth of *Fusarium proliferatum* and *F. subglutinans* on maize grains. This strain also effectively reduced the level of FUM synthesized by *F. subglutinans* by nearly 60% but did not have the same effect on FUM produced by *F. proliferatum*. Repečkienė et al. [50] demonstrated the effectiveness of the yeasts *Kluyveromyces marxianus*, *Metschnikowia pulcherrima,* and *Geothrix fermentans* in removing mycotoxins from wheat flour and feed. All the strains they tested eliminated 100% of aflatoxin and caused an 86.7% to 100% reduction in the production of ZEA. The content of DON in the flour and the feed was also significantly lowered. Taheur et al. [51] confirmed the ability of yeast isolated from kefir to remove mycotoxins previously added to YPD culture medium and milk. The strains they studied removed small amounts of OTA (6%) and aflatoxin B1 (AFB_1_) (8%) and up to 44% of ZEA from the medium. Better results were obtained by adding the yeast *Kazakhstania servazzii* to milk. This species reduced the amounts of OTA, AFB_1_, and ZEA by 74%, 62%, and 95%, respectively.

The decontamination of mycotoxins by microbial binding is a very diverse process that depends on the strain, the physiological state of the cells, the initial concentration of the toxin, and environmental conditions. The main mechanisms that yeast uses to reduce the occurrence of mycotoxins are adsorption to the cell surface, biodegradation, and inhibition of toxin biosynthesis [52]. Many authors have described cell surface adsorption in live yeast and yeast inactivated by high temperature or acid. It has even been shown that mycotoxins can be bound by prepared cell walls [53,54]. Literature data show that some yeasts also have the ability to biodegrade mycotoxins into less toxic or non-toxic metabolites. Vekiru et al. [55] demonstrated the possibility of converting ZEA into a non-estrogenic ZOM-1 product by *Trichosporon mycotoxinovorans*.

## 4. Materials and Methods

### 4.1. Microorganisms

Three phytopathogenic fungi, *F. culmorum, F. graminearum, F. poae,* and three yeast isolates, *C. shehatae* (C13)*, C. fluviatilis* (C14) and *C. tropicalis* (C28) were obtained from the Culture Collection of the Department of Biotechnology, Microbiology and Human Nutrition, University of Life Sciences in Lublin, Poland. Four yeast strains were isolated from the environment: *R. glutinis* (E20) and *C. carnescens* (E22) from ears of organic wheat, *C. saturnus* (K10) from conventionally grown wheat roots, and *M. guilliermondii* (K2) from conventionally grown oat roots.

The cultures of fungi and yeasts were kept fresh and viable by periodical transfers on malt extract agar (BTL, Lodz, Poland) medium under aseptic conditions throughout the study. They were stored at 4 °C.

### 4.2. Influence of Yeasts on Mycotoxin Production in Wheat Grain

#### 4.2.1. Yeast and Fungus Co-Culture on Wheat Grain

Portions of 30 g of wheat grain were weighed into 100 mL conical flasks, 5 mL of distilled water was added into each flask, and the vessels were autoclaved at 121 °C for 21 min. To the sterile grains, volumes of 5 mL of sterile distilled water were added, and then each portion of grain was inoculated with 1 mL of a suspension of *F. culmorum, F. graminearum,* or *F. poae* spores (5 × 10^6^ spores/mL), and 5 mL of a suspension one of the test yeasts (5 × 10^8^ cfu/mL). Control samples were flasks with grain inoculated with 1 mL of fungal spores and 10 mL of sterile distilled water. Samples were incubated for 14 days at 28 °C. The experiment was performed in triplicate.

#### 4.2.2. Determination of Mycotoxin Contents in Wheat Grains

After 14 days of incubation of *F. culmorum, F. graminearum,* and *F. poae* with the test yeasts on wheat grain (the experiment described in Section 4.2.1), we assessed the contents of the mycotoxins ZEA, DON, and NIV produced by those fungi. The grain samples were dried in a fume hood and milled in a laboratory ultra-centrifugal mill.

For the determination of ZEA, 5 g of plant material was homogenized for 3 min with the addition of 25 mL of acetonitrile: water (90:10, *v*/*v*). ZEA was extracted on Zearal Test columns according to the method described by Goliński et al. [56]. The eluate was evaporated to dryness at 40 °C under a nitrogen stream. The extracts were then dissolved in 500 µL of acetonitrile: water: methanol (46:46:8, *v*/*v*/*v*) and homogenized in an ultrasonic bath, filtered through 0.2 µm porosity filters The quantification of ZEA in the samples was carried out by injection of an aliquot—100 µL of the solution—into a Waters 2695 chromatograph (Milford, MA, USA) with a Waters 2475 Multiλ Fluorescence Detector (λex = 274 nm, λem = 440 nm) and a Waters 2996 Photodiode Array Detector. Chromatographic separation was performed on a Nova Pak C18 column, 3.9 × 150 mm, 4 µm (Waters Corp., Milford, MA, USA). The mobile phase was acetonitrile: water: methanol (46:46:8, *v*/*v*/*v*), at a flow rate of 0.5 mL/min. ZEA was identified by comparing the retention times of the samples with the mycotoxin standard. Quantification was performed by the external standard method using the peak areas and a calibration curve. A Photodiode Array Detector was used to confirm the presence of ZEA based on the characteristic spectrum. The quantification limit was 1.0 mg/kg. The experiment was performed in triplicate.

Group B trichothecenes were extracted from a sample of plant material, according to the method described by Perkowski et al. [57]. DON and NIV were analysed as trimethylsilyl derivatives using an external standard, on a Varian 450-GC gas chromatograph (Agilent Tech., Santa Clara, C, USACity, State if Canada/USA, Country) with a Varian 320-MS mass detector. Trimethylsilyl derivatives were prepared by reaction with 100 µL of a TMSI/TMCS (trimethylsilyl imidazole/trimethylchlorosilane) mixture (100:1, *v*/*v*). One μL samples were injected into the dosing chamber at 280 °C without splitting the stream, at a separator temperature of 290 °C. The analysis of the trichothecenes was performed in the MRM mode, and the retention time was 13.16 min for DON and 14.72 min for NIV. The helium flow rate was 0.7 mL/min. The mycotoxin quantification limit was 1 mg/kg. The experiment was performed in triplicate.

### 4.3. Determination of Biogenic Amines

The safety of use of the investigated yeast isolates in food technology was checked and tested by determining whether they produced biogenic amines, according to the method provided by Aslankoohi et al. [43]. For this purpose, a suspension of the test yeast with a density of 5 × 10^8^ cfu/mL was inoculated in Petri dishes with YPD medium (10 g yeast extract, 20 g mycological peptone, 20 g glucose, 20 g agar per 1 l of distilled water), supplemented with bromocresol red (0.006%), and a mixture of amino acids with a total mass concentration of 1%. The amino acid mixture consisted of equal amounts of tyrosine, histidine, phenylalanine, leucine, tryptophan, arginine, and lysine. The spotted medium was incubated at 28 °C for 72 h. The control was a medium without amino acids. The experiment was carried out in triplicate. Biogenic-amine-producing yeasts were identified by a purple halo around their colonies.

### 4.4. Determination of Mycotoxin Contents in Model Bread

The study was conducted to determine the effect of yeast inoculum on the concentration of *Fusarium* mycotoxins in model bread baked from wheat grain contaminated with *F. culmorum, F. graminearum,* and *F. poae* mycelium. In the laboratory baking trial, the yeasts that did not produce biogenic amines were used.

To contaminate the grains, 1 kg of wheat was added to each of the three 2000 mL flasks used in this experiment; then 20 mL of distilled water was added, and the flasks were autoclaved at 121 °C for 21 min. After the flasks had cooled down, they were inoculated with mycelial fragments of one of the three pathogens *F. culmorum, F. graminearum,* or *F. poae*. The flasks were left to stand for 10 days at room temperature in order for the fungi to produce *Fusarium* mycotoxins. After this time, samples were prepared for grinding. The grain was evenly distributed and dried in a fume hood until a humidity of 15% was obtained. Then, it was milled in a Quadrumat Junior laboratory mill and divided into two milling fractions: flour and bran.

The laboratory baking trial was performed using the traditional technique according to the methodology provided by Szwedziak et al. [58] and Soboleva et al. [38], with modifications. One loaf of bread was baked with 100 g of flour, 100 mL of warm water, 10 g of fresh baker’s yeast, 3 g of sugar, 1.5 g of salt, and a 2% (*v*/*v*) addition of a suspension of the test yeast *C. shehatae* (C13), *C. fluviatilis* (C14), *M. guilliermondii* (K2), *C. saturnus* (K10), *R. glutinis* (E20) with a density of 5 × 10^8^ cfu/mL. The control was the same dough without the addition of the yeast suspension. All ingredients were mixed, the dough was formed and set aside for 45 min at 35 °C. The loaves were baked at 200 °C for 20 min. After baking, the loaves were placed on trays and left for 7 days until they became stale; they were then dried in an oven at 100 °C for 1 h and ground in a laboratory ultra-centrifugal mill. The experiment was performed in triplicate [59].

The contents of the mycotoxins ZEA, DON, and NIV were determined in the bread using the methodology described in Section 4.2.2. The experiment was duplicated.

### 4.5. Statistical Analysis

A statistical analysis of the results was carried out using Statistica 13.3 (Statsoft, Cracow, Poland) and Excel 2016 (Microsoft, Washington, DC, USA). To compare the results, a one-way or a multi-factor analysis of variance (ANOVA) was carried out, after first finding the normality of the distribution of the dependent variable in the compared groups, equal to the variances. The significance of differences between the individual group means was determined using Tukey’s post hoc test. All statistical hypotheses were verified at the significance level of *p* < 0.05.

## 5. Conclusions

The demand for natural, less processed products without the addition of chemical compounds, induces the search for and the development of new methods of preserving food and eliminating harmful microorganisms and their metabolites from food products. In practical terms, the results reported in this study indicate that the selected yeast isolates are a promising alternative in bakeries as components of new starter cultures to lower the content of *Fusarium* mycotoxins. Yeasts that do not produce biogenic amines and reduce the production of NIV, ZEA (above 35%), and DON (above 33%) in bread can improve its safety for the consumer. There is a great potential for non-conventional yeast strains to be used in the bakery industry in order to improve product quality and reduce chemical food additives. Further research is needed to demonstrate the Qualified Presumption of Safety (QPS) or Generally Regarded as Safe (GRAS) status of most of these microorganisms, such that they can be used in the food industry. This is important, because the current limitation in applications of non-conventional yeasts is that they are less studied, and their genetic architectures and pathways are less understood.

## Figures and Tables

**Table 1:** Mycotoxin contents in wheat grain after 14 days of incubation (28 °C) of *F. culmorum* with the test yeasts.

Test Yeasts	Content of Mycotoxins Produced by *F. culmorum* in Wheat Grain (μg/kg) During Co-Culture with Yeasts		
	DON	NIV	ZEA
*Candida shehatae* C13	815.0 ^d^ * ± 24.1	654.3 ^e^ ± 14.6	162.5 ^e^ ± 9.6
*Candida fluviatilis* C14	427.5 ^c^ ± 19.6	405.0 ^d^ ± 12.7	113.0 ^d^ ± 10.2
*Candida tropicalis* C28	1168.7 ^ab^ ± 34.9	802.1 ^b^ ± 13.8	216.0 ^b^ ± 9.1
*Meyerozyma guilliermondii* K2	1168.6 ^ab^ ± 33.5	900.7 ^ac^ ± 22.8	238.1 ^ac^ ± 9.8
*Cyberlindnera saturnus* K10	1218.3 ^a^ ± 22.5	915.9 ^a^ ± 13.5	246.0 ^a^ ± 10.1
*Rhodotorula glutinis* E20	1186.2 ^a^ ± 39.1	909.7 ^a^ ± 12.5	247.8 ^a^ ± 8.8
*Cryptococcus carnescens* E22	1116.0 ^b^ ± 41.9	769.4 ^bc^ ± 13.2	223.6 ^bc^ ± 6.4
Control	1184.6 ^a^ ± 38.7	917.4 ^a^ ± 23.5	241.8 ^a^ ± 10.0

* Mean values with the same letters (a–e) within the individual columns do not differ significantly at *p* < 0.05 according to Tukey’s post hoc test ± standard deviation.

**Table 2:** Mycotoxin contents in wheat grain after 14 days of incubation (28 °C) of *F. graminearum* with the test yeasts.

Test Yeasts	Content of Mycotoxins Produced by *F. graminearum* in Wheat Grain (μg/kg) During Co-Culture with Yeasts		
	DON	NIV	ZEA
*Candida shehatae* C13	300.4 ^e^ * ± 30.4	124.9 ^e^ ± 9.3	68.1 ^a^ ± 8.0
*Candida fluviatilis* C14	382.2 ^ab^ ± 14.4	159.2 ^a^ ± 11.2	71.3 ^a^ ± 7.9
*Candida tropicalis* C28	352.5 ^a^ ± 16.7	173.9 ^ab^ ± 15.2	69.5 ^a^ ± 8.2
*Meyerozyma guilliermondii* K2	416.2 ^c^ ± 11.1	194.6 ^c^ ± 8.3	81.7 ^a^ ± 8.6
*Cyberlindnera saturnus* K10	400.2 ^bc^ ± 9.2	183.6 ^bc^ ± 10.2	74.6 ^a^ ± 6.5
*Rhodotorula glutinis* E20	504.5 ^d^ ± 19.6	227.8 ^d^ ± 10.6	105.5 ^b^ ± 5.6
*Cryptococcus carnescens* E22	535.6 ^d^ ± 10.7	244.5 ^d^ ± 8.9	98.2 ^b^± 8.7
Control	1382.3 ^f^ ± 16.4	932.2 ^f^ ± 27.5	273.3 ^c^ ± 6.1

* Mean values with the same letters (a–f) within the individual columns do not differ significantly at *p* < 0.05 according to Tukey’s post hoc test; ± standard deviation.

**Table 3:** Mycotoxin contents in wheat grain after 14 days of incubation (28 °C) of *F. poae* with the test yeasts.

Test Yeasts	Content of Mycotoxins Produced by *F. poae* in Wheat Grain (μg/kg) During Co-Culture with Yeasts		
	DON	NIV	ZEA
*Candida shehatae* C13	603.2 ^d^ * ± 14.7	738.1 ^a^ ± 16.6	50.1 ^b^ ± 7.4
*Candida fluviatilis* C14	920.6 ^a^ ± 22.3	751.3 ^a^ ± 14.7	83.7 ^d^ ± 5.6
*Candida tropicalis* C28	307.6 ^c^ ± 19.5	425.0 ^d^ ± 13.8	24.8 ^a^ ± 3.7
*Meyerozyma guilliermondii* K2	194.1 ^b^ ± 31.5	287.5 ^c^ ± 13.7	16.8 ^a^ ± 3.1
*Cyberlindnera saturnus* K10	664.4 ^e^ ± 32.2	869.0 ^b^ ± 17.8	58.4 ^b^ ± 5.1
*Rhodotorula glutinis* E20	919.3 ^a^ ± 18.9	934.1 ^e^ ± 14.9	108.3 ^c^ ± 6.0
*Cryptococcus carnescens* E22	955.0 ^a^ ± 25.4	874.0 ^b^ ± 13.4	102.3 ^c^ ± 7.3
Control	924.6 ^a^ ± 27.2	971.3 ^f^ ± 32.1	106.0 ^c^ ± 9.2

* Mean values with the same letters (a–f) within the individual columns do not differ significantly at *p* < 0.05 according to Tukey’s post hoc test ± standard deviation.

**Table 4:** The contents of mycotoxins in model bread obtained from wheat grain inoculated with *F. culmorum, F. graminearum* and *F. poae* and the test yeasts.

Test Yeasts	Mycotoxins Content in Model Bread (µg/kg)		
	DON	NIV	ZEA
*Candida shehatae* C13	1154.0 ^b^ * ± 24.6	760.1 ^e^± 8.4	175.4 ^d^ ± 3.5
*Candida fluviatilis* C14	995.1 ^a^ ± 20.4	647.3 ^b^ ± 9.8	154.4 ^a^ ± 4.7
*Meyerozyma guilliermondii* K2	920.0 ^c^ ± 13.7	606.4 ^a^ ± 9.2	130.4 ^c^ ± 2.4
*Cyberlindnera saturnus* K10	1012.9 ^a^ ± 34.2	666.0 ^c^ ± 8.8	160.1 ^ab^ ± 3.1
*Rhodotorula glutinis* E20	1135.4 ^b^ ± 13.6	731.1 ^d^ ± 10.1	165.9 ^b^ ± 3.2
Control	1380.8 ^d^ ± 21.5	933.5 ^f^ ± 20.2	204.5 ^e^ ± 5.7

* Mean values with the same letters (a–f) within the individual columns do not differ significantly at *p* < 0.05 according to Tukey’s post hoc test ± standard deviation.

## Data Availability

The data presented in this study are available on request from the corresponding author.

## Sample Availability:

Non-conventional yeast strains used for analysis are available from the authors.
